# Epigenetic aging in older people living with HIV in Eswatini: a pilot study of HIV and lifestyle factors and epigenetic aging

**DOI:** 10.1186/s13148-024-01629-7

**Published:** 2024-02-26

**Authors:** Christian K. Dye, Haotian Wu, Gabriela L. Jackson, Altaye Kidane, Rejoice Nkambule, Nomthandazo G. Lukhele, Bongiwe Prudence Malinga, Rhinos Chekenyere, Wafaa M. El-Sadr, Andrea A. Baccarelli, Tiffany G. Harris

**Affiliations:** 1 Department of Environmental Health Sciences, Columbia University Mailman School of Public Health, 630 West 168th St. Room 16-416, New York, NY 10032 USA; 2 ICAP at Columbia University, Mailman School of Public Health, New York, NY USA; 3 Eswatini Ministry of Health, Mbabane, Eswatini; 4 Department of Epidemiology, Columbia University Mailman School of Public Health, New York, NY USA

**Keywords:** Epigenetics, Epigenetic aging, Biological aging, HIV, Epigenetic age acceleration, DunedinPACE, Epigenetic clock, Older people living with HIV, Africa, Eswatini

## Abstract

**Background:**

People living with HIV (PLHIV) on effective antiretroviral therapy are living near-normal lives. Although they are less susceptible to AIDS-related complications, they remain highly vulnerable to non-communicable diseases. In this exploratory study of older PLHIV (OPLHIV) in Eswatini, we investigated whether epigenetic aging (i.e., the residual between regressing epigenetic age on chronological age) was associated with HIV-related parameters, and whether lifestyle factors modified these relationships. We calculated epigenetic aging focusing on the Horvath, Hannum, PhenoAge and GrimAge epigenetic clocks, and a pace of biological aging biomarker (DunedinPACE) among 44 OPLHIV in Eswatini.

**Results:**

Age at HIV diagnosis was associated with Hannum epigenetic age acceleration (EAA) (*β*-coefficient [95% Confidence Interval]; 0.53 [0.05, 1.00], *p* = 0.03) and longer duration since HIV diagnosis was associated with slower Hannum EAA (− 0.53 [− 1.00, − 0.05], *p* = 0.03). The average daily dietary intake of fruits and vegetables was associated with DunedinPACE (0.12 [0.03, 0.22], *p* = 0.01). The associations of Hannum EAA with the age at HIV diagnosis and duration of time since HIV diagnosis were attenuated when the average daily intake of fruits and vegetables or physical activity were included in our models. Diet and self-perceived quality of life measures modified the relationship between CD4^+^ T cell counts at participant enrollment and Hannum EAA.

**Conclusions:**

Epigenetic age is more advanced in OPLHIV in Eswatini in those diagnosed with HIV at an older age and slowed in those who have lived for a longer time with diagnosed HIV. Lifestyle and quality of life factors may differentially affect epigenetic aging in OPLHIV. To our knowledge, this is the first study to assess epigenetic aging in OPLHIV in Eswatini and one of the few in sub-Saharan Africa.

**Supplementary Information:**

The online version contains supplementary material available at 10.1186/s13148-024-01629-7.

## Introduction

The advent of highly effective antiretroviral therapy (ART) has shifted the landscape of HIV from a deadly disease to a chronic condition, often associated with multi-morbidities [1]. Access to ART and viral load suppression is associated with decrease in morbidity and mortality [2, 3], which has resulted in an increase in life expectancy for people living with HIV (PLHIV), from 8 to 10 years from time of HIV diagnosis [4] to near-normal life expectancy compared to the general population [5, 6]. As a consequence of ART use, PLHIV have experienced decreases in AIDS-related complications, however, are now at a greater risk of developing age-related comorbidities, including cardiovascular diseases (CVDs) [7], type 2 diabetes (T2D) [8], neurocognitive disorders [9], and cancers [10]. This change is particularly relevant for sub-Saharan Africa, where HIV remains highly prevalent, particularly in Eswatini, the country with the highest prevalence in the world, at 27.2% among those age 15–49 years old [11]. With the expansion of HIV treatment in sub-Saharan Africa, PLHIV there are showing marked increases in life expectancy [12]. The number of older PLHIV (OPLHIV), defined as those ≥ 50 years of age, is growing worldwide [13] and with the number of OPLHIV increasing in sub-Saharan Africa at a more rapid pace compared to higher-income countries [14] has resulted in increased rates of non-AIDS age-related comorbidities [15]. This is particularly challenging for countries that have few programs for persons with non-communicable diseases [16].

At the interface between HIV and HIV-associated comorbidities are aging trajectories that may be dysregulated in OPLHIV. Although HIV-infected people are living longer lives, they are aging at a faster rate, which is a likely outcome of the physiological effects of HIV [17]. A significant portion of the ART-treated population display aging-associated phenotypes and morbidities at a higher rate and at a significantly younger age as compared to the general aging population [18], including frailty [19], reduced bone density [20], and age-related non-communicable diseases (CVDs, T2D, metabolic dysfunction, dementias, etc.) [21–24]. Although there is a paucity of data, with the increase in ART treatment access, similar trends have been observed in regions of sub-Saharan Africa [15, 25, 26]. These differences in the aging process and the earlier onset of age-related phenotypes among OPLHIV may arise from differences in biological aging, the accumulation of damage to tissues and cells throughout the body as the aging process proceeds across the life-course; a process that is affected by environmental influences (pollutants, chemicals, lifestyle behaviors, infections, etc.) [27, 28].

DNA methylation (DNAm), the addition of a methyl group to the 5′ position of cytosine at a cytosine-guanine dinucleotide (CpG), is an epigenetic modification involved in regulating cellular phenotype via gene regulation without changes to the genotype [29]. Epigenetic mechanisms, including DNAm, have gained considerable attention in biomedical research, given that these epigenetic markers are responsive to environmental influences (e.g., lifestyle factors, infections, chemical exposures, etc.) and can regulate underlying cellular functions via transcriptional regulation, and when dysregulated, may underlie disease pathogenesis. Importantly, microarray-based DNAm data have been used for the creation of epigenetic clocks, developed to predict chronological aging, such as the first-generation Horvath [30] and Hannum clocks [31] (i.e., epigenetic age), and the second-generation clocks, PhenoAge [32] and GrimAge [33], developed as biomarkers of biological age, and, finally, the pace of biological aging biomarker, DunedinPACE [34]. Higher epigenetic age, or biological age, as compared to chronological age, termed epigenetic age acceleration (EAA), has been associated with a wide range of age-related diseases and mortality [35–37]. Further, evidence suggests HIV infection may increase biological age by more than 5 years in untreated individuals [38], and despite ART and viral load suppression, which have been shown to slow biological aging, it remains relatively increased [39]. Although it is generally accepted that biological age is accelerated in OPLHIV, little is known about biological aging in OPLHIV in sub-Saharan Africa, a region disproportionately affected by HIV.

Additionally, in response to the growing epidemic of non-AIDS comorbidities in OPLHIV, strategies have been implemented that are targeted at mitigating, preventing, and managing non-communicable diseases in OPLHIV [40, 41]. However, in lower-middle-income countries, programs focused on the prevention of comorbidities in OPLHIV are limited for several reasons, including lack of resources, such as trained staff, integration of programs with health care systems, institutional support (e.g., government, department of health, facilities, etc*.*), and material resources (e.g., educational materials, equipment for HIV care, etc*.*) [42]. The limited programs to address non-communicable diseases in OPLHIV likely stem from—in addition to the lack of resources—HIV programs in sub-Saharan Africa that are primarily focused on getting individuals diagnosed, on ART treatment, and virally suppressed [43]. Even in uninfected populations, prevention of multimorbidity through interventions is inadequate due to economic constraints [44]. Investigating underlying features of HIV, and lifestyle and health behaviors that are associated with the aging process, will create better informed strategies to mitigate HIV-associated comorbidities in lower- and lower-middle-income countries.

In this study, we sought to investigate epigenetic aging among OPLHIV on ART in Eswatini by assessing whether HIV-related factors affect the aging process and to determine whether certain modifiable lifestyle and quality of life factors modify this relationship.

## Methods

### Study population

Participants for this study were recruited in an exploratory study of OPLHIV in Eswatini, detailed elsewhere [45]. In brief, a convenience sample of 50 PLHIV age ≥ 50 years receiving care at the Mankayane Government Hospital in Manzini region of Eswatini were recruited from October 2016 to January 2017. All participants provided written, informed consent. The study was approved by the Eswatini Directorate of Health Services/Public Health, the Eswatini Ethics Committee, and the Columbia University Irving Medical Center Institutional Review Board.

### Data collection

Quantitative interviews were conducted, and clinical and sociodemographic information were self-reported and abstracted from paper and electronic-based medical records for each participant. Trained staff collected sociodemographic information (e.g., age, education, employment/occupation, financial and medical support, etc*.*), medical history, HIV and sexually transmitted disease information, lifestyle behaviors (e.g., physical activity, smoking, diet), psychosocial behavior (e.g., depressive symptoms), contraceptive use, non-communicable disease history, injuries, violence, social support, and acute quality of life measurements.

To assess quality of life, the Short Form Health Survey (SF-36) was used [46], which is a 36-item questionnaire that measures self-reported quality of life by assessing responses across several functional domains, including vitality, physical function, bodily pain, general health perception, physical functioning, emotional functioning, social functioning, and mental health/emotional well-being with each response scored from 0 (low health status) to 100 (high health status). Self-reported dietary intake was measured using the CORE physical activity and diet questions from the WHO STEPwise approach to surveillance (STEPS) survey [47]. Average daily dietary intake was calculated based on the number of fruits and/or vegetable servings eaten on a typical day divided by the number of servings eaten in a week; and weekly dietary intake was the total of fruits and/or vegetables, calculated as the number of servings in a typical day multiplied by the number of days fruits/vegetables are eaten in a week. Physical activity was also assessed using the STEPwise approach. Here, we included self-reported physical activity as cumulative physical activity in any given week, which included work-related physical activity (number of days of vigorous- and moderate-intensity work-related activities × number of minutes on any given day of vigorous- and moderate-intensity work-related activities), travel-related physical activity (i.e., number of days per week spent biking or walking to work × number of minutes spent biking or walking to work), and leisure time physical activity (number of days of vigorous- and moderate-intensity leisure physical activity × the number of minutes on any given day of vigorous- and moderate-intensity leisure physical activity). Similarly, self-reported recreational/leisure time physical activity minutes were calculated by the number of days in a week multiplied by the number of minutes of moderate and vigorous physical activity. HIV-related factors included data that were either self-reported (age at HIV diagnosis, age at ART initiation) or abstracted from clinical charts (WHO HIV clinical stage at the most recent visit, CD4^+^ T cell count [cells/µL] at enrollment and ART initiation, viral load at most recent visit). The number of years since HIV diagnosis and number of years on ART were calculated as the difference between age at HIV diagnosis and age at ART initiation with the participant’s chronological age at most recent visit.

### DNA methylation preprocessing, normalization, and quantification

Each participant had dried blood spot (DBS) specimens obtained using a finger prick; five separate drops (75–80 µL each) of blood were collected on a Protein Saver card. DNAm was quantified from the DBS that were collected from 46 of the initially recruited 50 participants following standardized protocols; two participants had low-quality DNA collected, one participant was missing abstracted data, and one participant was missing key self-reported questionnaire data. Blood spots were purified for genomic DNA. DNA was bisulfite-converted and hybridized to Illumina’s Infinium MethylationEPIC BeadChip (850K; Illumina, Inc. San Diego, CA, USA), improving on the coverage, reproducibility, and accuracy from its predecessor the HumanMethylation450 BeadChip, and allowing for the quantification of DNAm at > 850,000 CpGs at single-nucleotide resolution across the genome, covering > 99% RefSeq genes. Raw DNAm IDAT files were generated and loaded into the R statistical environment (version 4.2.2) using the *minfi* package in R [48]. Initial quality control removed one sample whose mean detection *p*-value across all probes was unreliable (detection *p*-value ≥ 0.01). Next, normalization was performed using the *ENmix* package in R for background correction using a flexible exponential-normal mixture distribution along with a truncated normal distribution to reflect background noise, followed by a dye-bias correction method using RELIC, which corrects for dye-bias across the array between the two color channels of the 850K [49, 50]. Next, we performed inter-array normalization using the quantile normalization for methylation intensities between samples. One sample out of the 46 collected that lacked typical bimodal distribution was also excluded from downstream analyses. Next, we performed probe filtering to remove low-quality CpG probes across samples and removed CpG probes when detection *p*-values were not significant against the background (detection *p*-value > 0.01) in one or more individuals, resided on sex chromosomes, harbored known single-nucleotide polymorphisms (SNPs) in the probe sequence, and were cross-reactive. Finally, due to the differential abundance in type I and type II probes on the 850K, we performed probe-type bias adjustment using the regression on correlated probes (RCP) normalization method within the *ENmix* package, which recalibrates type II probes to nearby type I probes. From 45 participants, DNAm values (β-values) were thus generated for 763,250 CpGs, which is based on the ratio of the methylated CpG probe to the total methylation state of the probe (methylated + unmethylated probe) resulting in a DNAm proportion from 0.0 (unmethylated) to 1.0 (fully methylated). Due to potential confounding caused by differences in cell-type heterogeneity that underlies epigenetic variability in heterogeneous samples (e.g., blood spots, whole blood, etc*.*) [51, 52] coupled with changes in immune cell composition inherent to the aging process [53] and during HIV infection [54], we estimated cell-type proportion of each blood sample (CD4^+^ T cell, CD8^+^ T cell, NK cell, monocyte, B cell, neutrophil), using the Houseman’s projection method employed in the *estimateCellCounts* function in the *minfi* package [55] to include in our epigenetic aging analyses.

### Epigenetic clock calculation

In order to estimate participants’ epigenetic/biological age, we included the two first-generation epigenetic clocks, the pan-tissue Horvath clock [30] and the blood immune cell Hannum clock [31], and two second-generation clocks that are shown to predict age-related phenotypes and lifespan (i.e., biological age), including PhenoAge [32] and GrimAge [33], these second-generation clocks differ from the first-generation epigenetic clocks that were trained to predict chronological age (i.e., epigenetic age). Due to one participant’s Hannum clock estimated epigenetic age deviating by more than 50 years compared to chronological age, we removed this participant from further analyses. Epigenetic aging trajectories were defined as the residuals between regressing estimated epigenetic/biological age on chronological age. When the resulting residual was positive, participants’ epigenetic/biological age was considered accelerated as compared to their chronological age (i.e., EAA) and negative values represented epigenetic age deceleration (EAD). Finally, we employed the DunedinPACE biomarker, which is intended as a DNAm-based biomarker that measures the rate of biological aging per calendar year [34].

### Statistical analyses

Descriptive statistics for participants who had reliable DNAm data available (*n* = 44) were calculated as median (1st quartile, 3rd quartile) for continuous variables and counts (%) for categorical variables.

For all statistical analyses, we focused on the first-generation Horvath and Hannum clocks, the second-generation PhenoAge and GrimAge clocks, and the DunedinPACE biomarker of the pace of biological aging. Due to the missingness of data from those enrolled in this study, our analyses specifically focused on HIV-relevant clinical parameters, and lifestyle and perceived quality of life variables that were present in ≥ 70% of participants. To test whether HIV-related variables were associated with EAA in OPLHIV in Eswatini, in our primary analysis, we performed linear regression analyses between HIV-relevant variables as the predictor variables (number of years living with HIV since diagnosis [years], age at HIV diagnosis [years], number of years on ART [years], age at ART initiation [years], CD4^+^ T cell count at enrollment [cells/µL], CD4^+^ T cell count at ART initiation [cells/µL]) and epigenetic aging changes as the outcome variable (*i.e.*, residuals of regressing estimated epigenetic metrics on chronological age), adjusting for relevant variables selected a priori, including age (years), sex (male or female), educational attainment (none, primary school, secondary school, high school, tertiary school), smoking status (never or ever), and estimated immune cell-type composition calculated from DNAm data (CD4^+^ cells, CD8^+^ cells, NK cells, B cells, monocytes, and neutrophils), which we considered in our primary analysis as Model 1. Although evidence suggests cell-type composition may underlie the variability in DNAm-based measures of biological aging, we have included regression models without further adjustment for DNAm estimations of cell-type composition in Additional file 1.

To investigate whether health and lifestyle factors modified the relationships between HIV-related variables and epigenetic aging, we first utilized linear regression analyses to test the association between health and lifestyle factors (i.e., average daily dietary intake of fruits and vegetables, weekly dietary intake of fruits and vegetables, total physical activity, and SF-36) as the predictor variable and epigenetic aging as the outcome. Next, we employed three separate models to determine whether lifestyle and perceived health factors modified the relationship between HIV-related characteristics and epigenetic aging, including, (1) a model that adjusted for a priori-selected covariates (the model used in the previous analysis [Model 1]) and average daily dietary intake fruits/vegetable servings (Model 2); (2) Model 1 with further adjustment for self-reported quality of life based on the SF-36 questionnaire (Model 3); and (3) Model 1 with further adjustment for total physical activity (total minutes of moderate and vigorous work, travel/commute, and recreational/leisure activity in a week) (Model 4). Statistical significance was defined as *p* < 0.05. Finally, similar to our other analyses, we have included regression models without DNAm-based estimation of cell-type composition in Additional file 1. Linear regression *β*-estimates (95% Confidence Intervals [CIs]) were calculated in R (version 4.2.2), and graphical figures were created using Prism (version 8; Dotmatics, Boston, MA).

## Results

### Participant characteristics

Descriptive statistics of participants included in this analysis (*n* = 44) are provided in Table 1. The median age (interquartile range [IQR]) was 59 [54, 66] years, slightly more than half of the participants were female (24 participants [54%]), the average weight was 75 (66, 87) kg, and the highest education attainment for most participants was primary school (grades 1–7; 23 participants [52%]). Participants had a median age of 53 (47, 58) years when diagnosed with HIV and had been living with HIV since diagnosis for 7 (5, 9) years until the most recent visit when DNAm data were collected. The median age at ART initiation was 53 (47, 59) years old, and participants had been on ART for 6 (4, 8) years. Most of the participants (40 [91%]) were categorized in WHO stage 1 (asymptomatic) at the most recent visit. CD4^+^ T cell counts at enrollment was 629 (457, 837) cell/µL, and at ART initiation, it was 202 (106, 289) cells/µL. At the most recent visit, the majority of participants were virally suppressed, including 40 participants that had ideal viral suppression (< 200 copies/mL), which included 27 participants with lower than detectable virus, 7 with < 20 copies/mL, and 6 with < 115 copies/mL, while the remaining 4 were missing (data not shown). Nine participants (21%) reported missing ART at least once in the last month. The median SF-36 quality of life score was 69 (53, 90). Participants reported consuming 0.8 (0.6, 1.3) fruits and/or vegetables per day, and no participants met the WHO standard for vegetable and fruit servings per week. Participants had 855 (390, 2220) minutes of total physical activity per week, with the majority through their work and commuting. Sixteen participants (36%) were past smokers, and three participants are current smokers (7%).

**Table 1:** Participant characteristics from pilot study of OPLHIV in Eswatini included in DNA methylation analyses

Variable	Participants (*n* = 44)
Age (years) [median (IQR)]	59 (54, 66)
*Sex*	
Female	24 (54%)
Male	20 (46%)
Weight, at recent visit (kg)	75 (66, 87)
*Education*	
None	5 (11%)
Primary School Grade 1–7	23 (52%)
Secondary School Form 1–3	12 (27%)
High School Form 4–5	4 (9%)
Years Since HIV Diagnosis (years)	7 (5, 9)
Age at HIV Diagnosis (years)	53 (47, 58)
Age at ART Initiation (years)	53 (47, 59)
ART Duration (years)	6 (4, 8)
WHO Clinical Stage 1 of HIV, at recent visit	40 (91%)
CD4^+^ T Cell Count at Enrollment (cell/uL)	629 (457, 837)
CD4+ T Cell Count at ART Initiation (cells/uL)	202 (106, 289)
Missed ART At Least Once in a Month, answered yes	9 (21%)
HIV Viral Load < 200 copies/mL, at recent visit	40 (91%)
SF-36 Health Questionnaire (0–100 Scale)	69 (53, 90)
Average Servings of Fruits and/or Vegetables, daily	0.8 (0.6, 1.3)
Servings of Fruits and/or Vegetables, weekly	10 (7, 14)
Eat Processed Food High in Salt, answered always/often	19 (43%)
Physical Activity	
Total Physical Activity, week (min)	855 (390, 2220)
Recreational/Leisure Time Physical Activity of ≥ 30 min in a week, answered yes	2 (5%)
Smoked Tobacco Ever, answered yes	16 (36%)
*Estimated Immune Cell Composition (% of Total Composition)*	
CD4+ T Cell	7% (5, 10)
CD8+ T Cell	23% (18, 27)
Natural Killer Cell	6% (5, 9)
B Cell	8% (5, 9)
Monocyte	10% (9, 12)
Neutrophil	51% (44, 55)
*Epigenetic Age Metrics*	
Horvath Epigenetic Age (years)	60 (56, 67)
Hannum Epigenetic Age (years)	52 (45, 58)
PhenoAge Biological Age (years)	68 (63, 77)
GrimAge Biological Age (years)	56 (50, 61)
DunedinPACE (pace to ref. of 1.0)	1.2 (1.1, 1.3)

Continuous variables: Median (1st quartile, 3rd quartile); categorical variables: counts, *n* (%)

WHO clinical stage of HIV: stage 1 (asymptomatic) to stage 4 (AIDS)

SF-36 Health Questionnaire: 36-item health survey to assess overall quality of life; 0 (low) to 100 (high)

WHO recommends 35 servings of fruits and/or vegetables per week

Total physical activity calculated as the number of minutes of work-related (moderate, vigorous), commuting-related (bike, walk), and recreational/leisure time (moderate, vigorous) physical activity

Estimated immune cell composition calculated using the Houseman's projection method for DNA methylation data

*ART* antiretroviral therapy, *IQR* interquartile range, *WHO* World Health Organization

### HIV status and epigenetic age acceleration

**Fig. 1:**
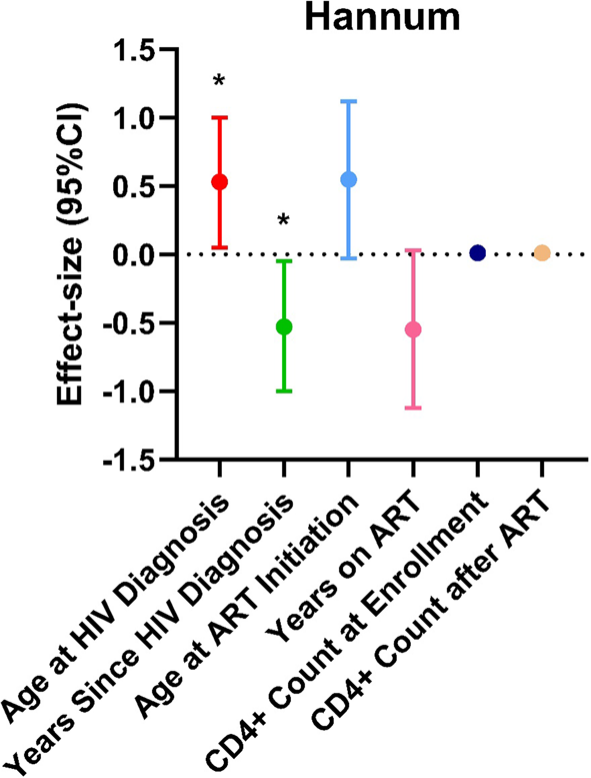
Effect sizes for association between clinical variables of HIV status and epigenetic aging from older people living with HIV (OPLHIV) (*N* = 44), Eswatini, 2016–17. Effect estimates (95% Confidence Interval [CI]) for the change in epigenetic age acceleration (EAA) calculated from dried blood spots from OPLHIV using HIV status-related variables, including: age at HIV diagnosis (red), years since HIV diagnosis (green), age at antiretroviral therapy (ART) initiation (light blue), years on ART (pink), CD4^+^ T cell count at enrollment (navy) and CD4^+^ T cell count at ART initiation (tan). Figure depicts change in Hannum EAA. Plots are adjusted for age, sex, educational attainment, past smoking status, and estimated cell-type composition (CD4^+^ T cell, CD8^+^ T cell, NK cell, B cell, monocyte, and neutrophil). Significance taken at *p* < 0.05, represented by *; *p* < 0.05 *

Descriptive statistics of epigenetic age calculated for each epigenetic clock and DunedinPACE are detailed in Table 1. Participants were generally biologically older when using the Horvath and PhenoAge clocks, having a median (IQR) age of 60 (56, 57) years and 68 (63, 77) years, respectively, as compared to a chronological age of 59 (54, 66) years. Participants were biologically younger using the Hannum (52 [45, 58] years) and GrimAge clocks (56 [50, 61] years) as compared to chronological age. Given the differences observed between epigenetic age and chronological age, we sought to determine whether HIV-related variables were associated with differences in epigenetic aging trajectories. Overall, we found no significant association between HIV-related variables with Horvath, PhenoAge, GrimAge or DunedinPACE (Additional file 1: Table S1; all *p* > 0.05). Using the Hannum epigenetic clock, we observed the age when participants were diagnosed with HIV was associated with a higher EAA (0.53 [0.05, 1.00], *p* = 0.03; Fig. 1; Additional file 1: Table S1) and the number of years since HIV diagnosis was associated with lower EAA (− 0.53 [− 1.00, − 0.05], *p* = 0.03; Fig. 1; Additional file 1: Table S1). Further, the age at which participants began ART treatments was approaching significance in its relationship with Hannum EAA (0.55 [-0.03, 1.12*], p* = 0.06) and the number of years on ARTs association with Hannum EAA was trending toward significance (− 0.55 [− 1.12, 0.03], *p* = 0.06).

### Health and lifestyle factors and HIV-associated epigenetic age acceleration

We next investigated whether quality of life and lifestyle behaviors were associated with epigenetic aging (Additional file 1: Table S2). We observed a significant association between the average daily intake of fruits and vegetables and DunedinPACE (0.12 [0.03, 0.22]; *p* = 0.01). There were no significant associations for the first-generation or second-generation clocks with lifestyle and quality of life characteristics (all *p* > 0.05) (Additional file 1: Table S2).

**Fig. 2:**
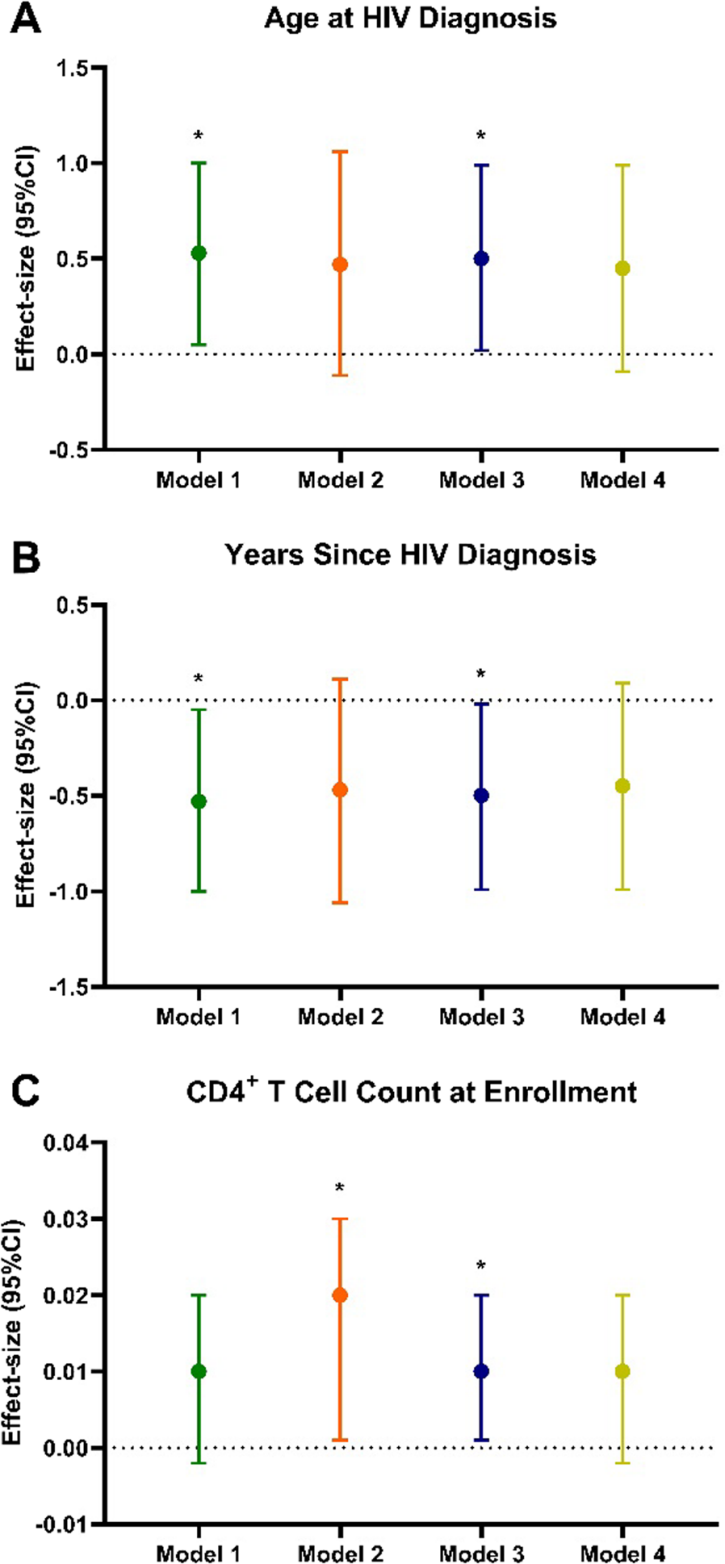
Effect sizes for the associations between HIV status variables and epigenetic aging adjusted for lifestyle and quality of life factors. Effect estimates (95% CI) for the change in **A** Hannum EAA by age at HIV diagnosis, **B** Hannum EAA by years since HIV diagnosis, and **C** Hannum EAA by CD4^+^ T cell counts at enrollment. Each plot represents effect estimates for four separate models: Model 1 (Green; covariates: age, sex, education level, past smoking status, and estimated cell-type composition); Model 2 (Orange; Model 1 + weekly dietary servings of fruits/vegetables); Model 3 (Navy; Model 1 + SF-36 questionnaire [perceived quality of life]); Model 4 (Green-Yellow; Model 1 + total physical activity [work-related moderate/vigorous + commuting + leisure/recreational moderate/vigorous activity] Significance taken at *p* < 0.05, represented by *; *p* < 0.05 *

Next, we sought to investigate whether health and lifestyle behaviors modified the relationship between HIV status and epigenetic aging trajectories (Fig. 2A–C; Additional file 1: Table S1). We found no notable effects of lifestyle factors on the associations between Horvath, GrimAge and DunedinPACE with clinical variables of HIV status (Additional file 1: Table S1). The association of age at HIV diagnosis with Hannum EAA in our primary model (Model 1) remained statistically significant after further adjustment for self-reported quality of life measurements derived from the SF-36 report (0.48 [0.04, 0.92], *p* = 0.03; Fig. 2A, Additional file 1: Table S1), which was similarly observed between Hannum EAA and the number of years since HIV diagnosis (− 0.48 [− 0.92, − 0.04], *p* = 0.03). However, when further adjusting our primary model for average daily intake of fruits and vegetables (Model 2) and physical activity (Model 4), we found the association between age at HIV diagnosis and Hannum EAA was attenuated and resulted in a non-significant association (Model 2: 0.47 [− 0.07, 1.00], *p* = 0.08; Model 4: 0.39 [− 0.08, 0.86], *p* = 0.10; Fig. 2A, Additional file 1: Table S1). Similarly, when adjusting for average daily intake of fruits and vegetables and physical activity, we found an attenuated association between the number of years since HIV diagnosis and Hannum EAA (Model 2: − 0.47 [− 1.06, 0.11], *p* = 0.10; Model 4: − 0.45 [− 0.99, 0.09], *p* = 0.10; Fig. 2B, Additional file 1: Table S1).

We also observed that CD4^+^ T cell counts at enrollment were associated with higher Hannum EAA in the model that included the average daily intake of fruits and vegetables (0.02 [0.001, 0.03], *p* = 0.03; Fig. 2C, Additional file 1: Table S1) or self-reported quality of life measurements (0.01 [0.001, 0.02], *p* = 0.03; Fig. 2C, Additional file 1: Table S1). Lastly, we found a negative association that was approaching significance between CD4^+^ T cell counts after ART initiation with Horvath EAA (− 0.03 [− 0.07, 0.004], *p* = 0.07; Additional file 1: Table S1) and PhenoAge EAA (− 0.04 [− 0.08, 0.004], *p* = 0.07; Additional file 1: Table S1) in the model that was further adjusted for average daily intake of fruits and vegetables.

## Discussion

Our results show that the age at HIV diagnosis and the duration since HIV diagnosis are associated with differences in epigenetic aging. HIV diagnosis at an older age was associated with higher epigenetic aging, and conversely, those living with HIV for a longer duration since HIV diagnosis displayed lower epigenetic aging. Additionally, our results showed that those who were diagnosed with HIV at an older age exhibited increased epigenetic aging. Prior to DNAm-based measures of epigenetic aging, several studies have reported increased incidence of age-related phenotypes and biological changes associated with aging in PLHIV, which occurred at a significantly younger age compared to non-infected counterparts [17, 56, 57]. This has been further established with the recent improvement in epigenetic clocks, with results showing that epigenetic and biological age is accelerated in PLHIV compared to HIV-uninfected individuals, although most of these studies were conducted in higher-income countries [38, 58]. In our study, we found epigenetic age was accelerated in OPLHIV in Eswatini as compared to their chronological age, which may reflect other reports suggesting higher epigenetic aging in PLHIV as compared to their non-infected counterparts [38, 58]. Whether epigenetic age in PLHIV is accelerated compared to non-infected individuals in Eswatini warrants further investigation. Finally, we found that lifestyle and self-reported measures of quality of life modified the relationship between HIV-related variables with epigenetic aging trajectories.

HIV infection has extensive effects on immune cellular activity and function, including, most notably, CD4^+^ cell depletion [59], and such changes in immune cell activity are characteristic of biological aging [60]. Even with early ART administration, immune cell functions are incompletely restored, and chronic adverse immune-related phenotypes persist [61, 62]. Low-grade, chronic inflammation is a significant feature of biological aging, termed by some as ‘inflammaging’, and it can have detrimental effects on physiology and cellular functions, and has been linked to age-related disease pathogenesis [63, 64]. Regarding the significant association between the age at HIV diagnosis and Hannum-derived epigenetic aging, having acquired HIV at an older age, when inflammaging is likely already running its course, may further exacerbate the inflammatory effects of HIV on an already inflammatory milieu observed in the elderly [65], which may underlie the rapid functional decline and vulnerability to non-communicable diseases observed in OPLHIV. Likewise, a recent meta-analysis suggests the time between HIV infection and diagnosis is around 3 years [66]; however, this has been performed in high- and upper-middle-income countries and whether it is similar in lower-middle income countries, such as Eswatini, is not known, and may, in fact, be longer. This is critical, given that the longer time intervals between infection and diagnosis—where diagnosis would come with immediate ART treatment—is indispensable not only for prevention of transmission but for long-term disease management and strategies to combat age-related morbidity and premature mortality. Left untreated, HIV can augment inflammation and has been shown to accelerate the aging process [67, 68]. We posit that the older age of diagnosis could encompass a long window between infection and treatment in which the virus can cause a myriad of detrimental effects. Contrary to higher epigenetic aging associating with being diagnosed with HIV at an older age, the slower epigenetic aging observed in those that have been living longer with HIV since diagnosis may be the result of the beneficial effects of early intervention and long-term adherence to ARTs on restoring the immune system and improving aging trajectories. Notably, epigenetic age was accelerated in PLHIV that have untreated HIV infection [68] and initiation of ART may decelerate epigenetic aging [69]. Taken together, it is possible that the older age at infection may augment an already pro-inflammatory milieu typical of the elderly or suggests a potentially longer duration of living with untreated HIV, which can also have effects on epigenetic aging trajectories through inducing chronic pro-inflammatory phenotypes, while timely initiation of ART may benefit aging trajectories by attenuating the effects of untreated HIV.

Despite the use of ART, the risk of developing comorbidities in OPLHIV remains relatively high, which is likely due to one or more factors (e.g., effects of HIV, ART, lifestyle behaviors, etc.) that influence the aging process. To address this, several interventions have been implemented to reduce HIV-associated comorbidities. For example, the PRECluDE Consortium, funded by the National Institute of Health, has modeled several interventions and clinical trials for PLHIV for CVD prevention [40] and the REPRIEVE phase 3 trial has shown marked reductions in CVD risk using pitavastatin calcium in PLHIV [70], although these efforts have yet to be implemented in sub-Saharan Africa. Mounting evidence suggests that health-promoting behaviors are linked to slower aging [71, 72]. Practical interventions have been developed for prevention and management of chronic diseases in OPLHIV, including physical activity and nutritional interventions [73, 74]. Thus, developing practical strategies to mitigate comorbidities in OPLHIV will be valuable in lower-middle-income countries where resources remain constrained. Here, we found lifestyle and quality of life measures may have a modifying role in the association between HIV-related factors and epigenetic aging trajectories.

In our study, we found the relationship between epigenetic age acceleration and the age at HIV diagnosis, and the number of years since diagnosis was modified by physical activity and diet. Prior evidence suggests moderate-intensity physical activity, including from occupational activity, can improve clinical indicators of immune function in HIV-infected patients and improve aging [75, 76], which is especially beneficial to older populations [77]. To our knowledge, there is no data on the association of dietary interventions and epigenetic aging trajectories in PLHIV. Previous evidence in HIV-uninfected individuals suggests that dietary interventions can have beneficial effects on biological aging, decelerating, and potentially reversing, biological aging, including interventions focused on higher quality foods and specific types of diets (e.g., Mediterranean Diet, DASH Diet, etc.) [78–83]. The reduction in epigenetic age acceleration in those that have been diagnosed at an older age could be derived from nutritional needs by OPLHIV to achieve proper immune function, reduce HIV-related complications and comorbidities associated with unhealthy eating patterns, which have all been linked to aging [13, 84]. The reduction in slower epigenetic aging that associated with the number of years since diagnosis when adjusting for diet we speculated could have been due to an interaction between long-term ART adherence and nutrition that could potentially affect drug metabolism, drug effectiveness, and the effects of ARTs in combination with certain foods on metabolic outcomes [85].

Next, we observed CD4^+^ T cell counts after enrollment into this study were significantly associated with higher Hannum-derived epigenetic age acceleration in models adjusted for dietary intake of fruits and vegetables or self-reported quality of life measurements derived from the SF-36. At the time of enrollment, participants were not yet treated for HIV, and untreated patients exhibit increased rates of epigenetic aging. Although higher levels of CD4^+^ T cell counts in HIV-infected individuals is an indicator of good health, in the early stages of HIV (i.e., acute infection) and prior to treatment, when patients may have CD4^+^ T cell counts that put them at a low risk for AIDS and AIDS-related complications, mortality still remains high compared to their uninfected counterparts [86], which potentially operates through excessive immune activation and chronic inflammation [67]. Evidence suggests nutrient quality both predicts CD4^+^ cell counts and is associated with a lower risk of mortality in PLHIV [87], and diet quality is associated with CD4^+^ T cell count [88, 89]. Finally, the positive relationship between CD4^+^ T cell counts at enrollment and Hannum epigenetic age acceleration when adjusted for self-reported quality of life, SF-36, could be due to those that perceive themselves as generally healthier having poor health-related behaviors. In one study, women who perceived their health as better also reported adverse alcohol drinking behaviors [90]. In HIV-infected participants, higher perceived health was associated with loss-to-follow for interventions and clinical care for HIV [91, 92]. Altogether, our findings suggest a differential role for lifestyle and quality of life measurements on epigenetic aging trajectories at different stages in HIV management and supports the utilization of practical interventions that would be beneficial for modifying biological aging trajectories and non-communicable disease risk for OPLHIV in lower- and lower-middle-income countries where costly interventions are not yet feasible.

While this study is, to our knowledge, the first to investigate epigenetic aging in OPLHIV in Eswatini, it has several limitations. First, this is an exploratory study and thus the findings are constrained by the limited sample of participants [45]. As our study used a cross-sectional design, we could not ascertain whether epigenetic aging trajectories changed over time, especially from early stages of infection (untreated) to viral load suppression. Likewise, these data were limited to the HIV-related factors included in the study. For example, the duration of time since HIV diagnosis is not reflective of time since HIV infection. Accumulating evidence suggests that the timeframe immediately after exposure until ART initiation may be important to biological aging trajectories and long-term health outcomes [68, 93]. Future research should examine changes in biological aging trajectories longitudinally, prior to ART initiation and potentially as early as when exposure occurred. Further, although Eswatini is a lower-middle income country, it is still constrained of resource, having high levels of poverty and unemployment, and collecting data from regions of the world that are still constrained of resources has its inherent problems, such as the robust collection of data across a range of variables over time, and future work should seek to recruit and retain participants in order to obtain a diversity of high-quality clinical, sociodemographic, and viral information over time. Finally, our study was performed exclusively in OPLHIV in Eswatini, thus, the findings may not be generalizable to other populations or regions in sub-Saharan Africa, to other lower-middle income countries, or, in general, to other OPLHIV. Although our study had these limitations, this exploratory study showed compelling evidence that may underlie aging trajectories in OPLHIV in a lower-middle-income country, which may provide a foundation for future studies to further explore the molecular mechanisms of aging in PLHIV in sub-Saharan Africa and other resource limited settings.

## Conclusions

We found evidence that HIV-related variables are associated with epigenetic aging in OPLHIV on stable ART in Eswatini, a country with limited resources in sub-Saharan Africa. We also showed that modifiable risk factors may be important in influencing epigenetic aging trajectories in OPLHIV, which may unveil potential avenues for practical interventions that may be relevant to slowing aging trajectories and non-communicable disease risk in OPLHIV in regions with limited resources. With increased access to ART in Eswatini, and other regions of sub-Saharan Africa, life expectancy of PLHIV will continue to increase with an expanding older population living with HIV, with increasing risk of non-communicable diseases. Developing practical interventions that are aligned with “healthy aging” will be critically important.

### Supplementary Information

**Additional file 1**. Supplementary Tables.

## Data Availability

The data that supports findings from this study are available upon reasonable request from TGH. The data are part of an on-going collaboration between ICAP and the Eswatini Ministry of Health, and thus, is not currently publicly available.
