# Supplementary material for: Epigenetic aging in older people living with HIV in Eswatini: a pilot study of HIV and lifestyle factors and epigenetic aging
Source: Clin Epigenetics. 2024 Feb 26;16:32. doi: 10.1186/s13148-024-01629-7 (PMC10895753; doi:10.1186/s13148-024-01629-7)
Supplement: Supplementary file 1 — Additional file 1. Supplementary Tables. [file 13148_2024_1629_MOESM1_ESM.docx]

| **Supplementary Table 1. Association between epigenetic aging and HIV status** | | | | | | | | | | | | | | | |
| --- | --- | --- | --- | --- | --- | --- | --- | --- | --- | --- | --- | --- | --- | --- | --- |
|  |  |  |  |  |  |  |  |  |  |  |  |  |  |  |  |
|  |  | Horvath Clock | |  | Hannum Clock | |  | PhenoAge Clock | |  | GrimAge Clock |  |  | DunedinPACE | |
| HIV Variable |  | β-Estimate (95% CI) | *p-*value |  | β-Estimate (95% CI) | *p-*value |  | β-Estimate (95% CI) | *p-*value |  | β-Estimate (95% CI) | *p-*value |  | β-Estimate (95% CI) | *p-*value |
| *Primary Models* |  |  |  |  |  |  |  |  |  |  |  |  |  |  |  |
| *Model 1* | | | | | | | | | | | | | | | |
| Age at HIV Diagnosis |  | 0.21 (-0.31,0.74) | 0.41 |  | **0.53 (0.05,1.00)** | **0.03** |  | 0.24 (-0.42,0.90) | 0.47 |  | -0.08 (-0.50,0.35) | 0.72 |  | -0.01 (-0.02,0.004) | 0.19 |
| Years Since HIV Diagnosis |  | -0.21 (-0.74,0.31) | 0.41 |  | **-0.53 (-1.00,-0.05)** | **0.03** |  | -0.24 (-0.90,0.42) | 0.47 |  | 0.08 (-0.35,0.50) | 0.72 |  | 0.01 (-0.004,0.02) | 0.19 |
| Age at ART Initiation |  | 0.02 (-0.61,0.65) | 0.95 |  | 0.55 (-0.03,1.12) | 0.06 |  | 0.12 (-0.67,0.91) | 0.76 |  | -0.15 (-0.65,0.36) | 0.56 |  | -0.005 (-0.02,0.01) | 0.47 |
| Years on ART |  | -0.02 (-0.65,0.61) | 0.95 |  | -0.55 (-1.12,0.03) | 0.06 |  | -0.12 (-0.91,0.67) | 0.76 |  | 0.15 (-0.36,0.65) | 0.56 |  | 0.005 (-0.01,0.02) | 0.47 |
| CD4+ Count at Enrollment |  | 0.002 (-0.01,0.01) | 0.77 |  | 0.01 (-0.002,0.02) | 0.11 |  | -0.001 (-0.01,0.01) | 0.90 |  | -0.002 (-0.01,0.01) | 0.68 |  | 0.00 (-0.00,0.00) | 0.92 |
| CD4+ Count after ART |  | 0.001 (-0.02,0.02) | 0.91 |  | 0.01 (-0.01,0.02) | 0.45 |  | -0.02 (-0.04,0.005) | 0.12 |  | 0.001 (-0.01,0.01) | 0.84 |  | 0.00 (-0.00,0.00) | 0.58 |
| *Model 2* |  |  |  |  |  |  |  |  |  |  |  |  |  |  |  |
| Age at HIV Diagnosis |  | 0.41 (-0.36,1.18) | 0.28 |  | 0.47 (-0.11,1.06) | 0.10 |  | 0.18 (-0.74,1.11) | 0.68 |  | -0.01 (-0.71,0.68) | 0.97 |  | -0.005 (-0.02,0.01) | 0.46 |
| Years Since HIV Diagnosis |  | -0.41 (-1.18,0.36) | 0.28 |  | -0.47 (-1.06,0.11) | 0.10 |  | -0.18 (-1.11,0.74) | 0.68 |  | 0.01 (-0.68,0.71) | 0.97 |  | 0.005 (-0.01,0.02) | 0.46 |
| Age at ART Initiation |  | 0.21 (-0.83,1.25) | 0.68 |  | 0.41 (-0.39,1.22) | 0.29 |  | 0.28 (-0.94,1.49) | 0.64 |  | -0.08 (-1.00,0.83) | 0.85 |  | 0.002 (-0.02,0.02) | 0.85 |
| Years on ART |  | -0.21 (-1.25,0.83) | 0.68 |  | -0.41 (-1.22,0.39) | 0.29 |  | -0.28 (-1.49,0.94) | 0.64 |  | 0.08 (-0.83,1.00) | 0.85 |  | -0.002 (-0.02,0.02) | 0.85 |
| CD4+ Count at Enrollment |  | 0.01 (-0.01,0.03) | 0.36 |  | **0.02 (0.001,0.03)** | **0.03** |  | 0.01 (-0.01,0.03) | 0.34 |  | -0.01 (-0.03,0.01) | 0.24 |  | -0.00 (-0.00,0.00) | 0.93 |
| CD4+ Count after ART |  | -0.03 (-0.07,0.004) | 0.07 |  | -0.02 (-0.06,0.02) | 0.20 |  | -0.04 (-0.08,0.004) | 0.07 |  | -0.01 (-0.05,0.02) | 0.40 |  | -0.00 (-0.001,0.00) | 0.10 |
| *Model 3* | | | | | | | | | | | | | | | |
| Age at HIV Diagnosis |  | 0.23 (-0.31,0.76) | 0.39 |  | **0.50 (0.02,0.99)** | **0.04** |  | 0.27 (-0.4,0.94) | 0.42 |  | -0.09 (-0.52,0.34) | 0.68 |  | -0.01 (-0.02,0.004) | 0.22 |
| Years Since HIV Diagnosis |  | -0.23 (-0.76,0.31) | 0.39 |  | **-0.50 (-0.99,-0.02)** | **0.04** |  | -0.27 (-0.94,0.4) | 0.42 |  | 0.09 (-0.34,0.52) | 0.68 |  | 0.01 (-0.004,0.02) | 0.22 |
| Age at ART Initiation |  | 0.04 (-0.61,0.69) | 0.90 |  | 0.51 (-0.08,1.10) | 0.09 |  | 0.17 (-0.64,0.97) | 0.67 |  | -0.17 (-0.69,0.34) | 0.50 |  | -0.004 (-0.02,0.01) | 0.53 |
| Years on ART |  | -0.04 (-0.69,0.61) | 0.90 |  | -0.51 (-1.1,0.08) | 0.09 |  | -0.17 (-0.97,0.64) | 0.67 |  | 0.17 (-0.34,0.69) | 0.50 |  | 0.004 (-0.01,0.02) | 0.53 |
| CD4+ Count at Enrollment |  | 0.002 (-0.01,0.01) | 0.77 |  | **0.01 (0.001,0.02)** | **0.03** |  | -0.002 (-0.02,0.01) | 0.83 |  | -0.001 (-0.01,0.01) | 0.82 |  | 0.00 (-0.00,0.00) | 0.99 |
| CD4+ Count after ART |  | 0.001 (-0.02,0.02) | 0.91 |  | 0.01 (-0.01,0.02) | 0.45 |  | -0.02 (-0.04,0.004) | 0.12 |  | 0.001 (-0.01,0.01) | 0.84 |  | 0.00 (-0.000,0.00) | 0.59 |
| *Model 4* | | | | | | | | | | | | | | | |
| Age at HIV Diagnosis |  | 0.06 (-0.53,0.65) | 0.84 |  | 0.45 (-0.09,0.99) | 0.10 |  | 0.20 (-0.56,0.96) | 0.59 |  | 0.12 (-0.34,0.58) | 0.60 |  | -0.01 (-0.02,0.005) | 0.23 |
| Years Since HIV Diagnosis |  | -0.06 (-0.65,0.53) | 0.84 |  | -0.45 (-0.99,0.09) | 0.10 |  | -0.2 (-0.96,0.56) | 0.59 |  | -0.12 (-0.58,0.34) | 0.60 |  | 0.01 (-0.005,0.02) | 0.23 |
| Age at ART Initiation |  | -0.18 (-0.86,0.49) | 0.58 |  | 0.44 (-0.2,1.07) | 0.17 |  | 0.04 (-0.84,0.92) | 0.92 |  | 0.04 (-0.5,0.57) | 0.89 |  | -0.004 (-0.02,0.01) | 0.56 |
| Years on ART |  | 0.18 (-0.49,0.86) | 0.58 |  | -0.44 (-1.07,0.2) | 0.17 |  | -0.04 (-0.92,0.84) | 0.92 |  | -0.04 (-0.57,0.5) | 0.89 |  | 0.004 (-0.01,0.02) | 0.56 |
| CD4+ Count at Enrollment |  | 0.001 (-0.01,0.01) | 0.84 |  | 0.01 (-0.002,0.02) | 0.12 |  | -0.001 (-0.02,0.01) | 0.86 |  | -0.001 (-0.01,0.01) | 0.75 |  | 0.00 (-0.00,0.00) | 0.90 |
| CD4+ Count after ART |  | 0.002 (-0.01,0.02) | 0.82 |  | 0.01 (-0.01,0.03) | 0.37 |  | -0.02 (-0.04,0.01) | 0.15 |  | 0.00 (-0.01,0.01) | 1.00 |  | 0.00 (-0.00,0.00) | 0.64 |
| *Secondary Models* |  |  |  |  |  |  |  |  |  |  |  |  |  |  |  |
| *Model 1* | | | | | | | | | | | | | | | |
| Age at HIV Diagnosis |  | 0.07 (-0.50,0.64) | 0.80 |  | **0.47 (0.03,0.91)** | **0.04** |  | 0.22 (-0.41,0.86) | 0.48 |  | -0.03 (-0.51,0.45) | 0.89 |  | -0.004 (-0.02,0.01) | 0.50 |
| Years Since HIV Diagnosis |  | -0.07 (-0.64,0.50) | 0.80 |  | **-0.47 (-0.91,-0.03)** | **0.04** |  | -0.22 (-0.86,0.41) | 0.48 |  | 0.03 (-0.45,0.51) | 0.89 |  | 0.004 (-0.01,0.02) | 0.50 |
| Age at ART Initiation |  | -0.19 (-0.83,0.45) | 0.55 |  | 0.44 (-0.07,0.94) | 0.09 |  | 0.001 (-0.72,0.72) | 1.00 |  | -0.19 (-0.73,0.35) | 0.49 |  | -0.005 (-0.02,0.01) | 0.51 |
| Years on ART |  | 0.19 (-0.45,0.83) | 0.55 |  | -0.44 (-0.94,0.07) | 0.09 |  | -0.001 (-0.72,0.72) | 1.00 |  | 0.19 (-0.35,0.73) | 0.49 |  | 0.005 (-0.01,0.02) | 0.51 |
| CD4+ Count at Enrollment |  | -0.005 (-0.01,0.004) | 0.31 |  | 0.003 (-0.004,0.01) | 0.44 |  | 0.00 (-0.01,0.01) | 0.99 |  | -0.001 (-0.01,0.01) | 0.71 |  | 0.00 (-0.00,0.00) | 0.92 |
| CD4+ Count after ART |  | -0.003 (-0.02,0.01) | 0.62 |  | 0.01 (-0.01,0.02) | 0.22 |  | -0.004 (-0.02,0.02) | 0.67 |  | 0.01 (-0.01,0.02) | 0.24 |  | 0.00 (-0.00,0.001) | 0.35 |
| *Model 2* |  |  |  |  |  |  |  |  |  |  |  |  |  |  |  |
| Age at HIV Diagnosis |  | 0.14 (-0.60,0.88) | 0.69 |  | 0.47 (-0.07,1.00) | 0.08 |  | 0.24 (-0.55,1.03) | 0.54 |  | 0.17 (-0.47,0.82) | 0.59 |  | 0.002 (-0.01,0.02) | 0.78 |
| Years Since HIV Diagnosis |  | -0.14 (-0.88,0.60) | 0.69 |  | -0.47 (-1.00,0.07) | 0.08 |  | -0.24 (-1.03,0.55) | 0.54 |  | -0.17 (-0.82,0.47) | 0.59 |  | -0.002 (-0.02,0.01) | 0.78 |
| Age at ART Initiation |  | -0.18 (-1.10,0.74) | 0.69 |  | 0.45 (-0.24,1.13) | 0.19 |  | 0.19 (-0.80,1.17) | 0.70 |  | 0.02 (-0.78,0.82) | 0.96 |  | 0.01 (-0.01,0.02) | 0.49 |
| Years on ART |  | 0.18 (-0.74,1.10) | 0.69 |  | -0.45 (-1.13,0.24) | 0.19 |  | -0.19 (-1.17,0.80) | 0.70 |  | -0.02 (-0.82,0.78) | 0.96 |  | -0.01 (-0.02,0.01) | 0.49 |
| CD4+ Count at Enrollment |  | -0.01 (-0.02,0.01) | 0.31 |  | 0.003 (-0.01,0.01) | 0.50 |  | 0.01 (-0.01,0.02) | 0.33 |  | -0.001 (-0.01,0.01) | 0.79 |  | 0.00 (-0.00,0.00) | 0.43 |
| CD4+ Count after ART |  | **-0.02 (-0.04,-0.001)** | **0.04** |  | 0.001 (-0.02,0.02) | 0.93 |  | -0.01 (-0.05,0.02) | 0.48 |  | 0.01 (-0.02,0.03) | 0.67 |  | -0.00 (-0.001,0.00) | 0.93 |
| *Model 3* | | | | | | | | | | | | | | | |
| Age at HIV Diagnosis |  | 0.07 (-0.51,0.65) | 0.80 |  | **0.48 (0.04,0.92)** | **0.03** |  | 0.21 (-0.42,0.84) | 0.51 |  | -0.04 (-0.52,0.45) | 0.87 |  | -0.005 (-0.02,0.01) | 0.44 |
| Years Since HIV Diagnosis |  | -0.07 (-0.65,0.51) | 0.80 |  | **-0.48 (-0.92,-0.04)** | **0.03** |  | -0.21 (-0.84,0.42) | 0.51 |  | 0.04 (-0.45,0.52) | 0.87 |  | 0.005 (-0.01,0.02) | 0.44 |
| Age at ART Initiation |  | -0.19 (-0.84,0.46) | 0.56 |  | 0.43 (-0.08,0.95) | 0.09 |  | 0.003 (-0.71,0.72) | 0.99 |  | -0.19 (-0.73,0.36) | 0.49 |  | -0.005 (-0.02,0.01) | 0.49 |
| Years on ART |  | 0.19 (-0.46,0.84) | 0.56 |  | -0.43 (-0.95,0.08) | 0.09 |  | -0.003 (-0.72,0.71) | 0.99 |  | 0.19 (-0.36,0.73) | 0.49 |  | 0.005 (-0.01,0.02) | 0.49 |
| CD4+ Count at Enrollment |  | -0.005 (-0.01,0.005) | 0.31 |  | 0.003 (-0.004,0.01) | 0.46 |  | -0.005 (-0.01,0.01) | 0.96 |  | -0.001 (-0.01,0.01) | 0.72 |  | 0.00 (-0.00,0.00) | 0.87 |
| CD4+ Count after ART |  | 0.001 (-0.02,0.01) | 0.58 |  | 0.01 (-0.01,0.02) | 0.20 |  | -0.01 (-0.03,0.01) | 0.34 |  | 0.01 (-0.01,0.02) | 0.23 |  | 0.00 (-0.00,0.00) | 0.53 |
| *Model 4* | | | | | | | | | | | | | | | |
| Age at HIV Diagnosis |  | -0.13 (-0.72,0.47) | 0.67 |  | 0.39 (-0.08,0.86) | 0.10 |  | 0.29 (-0.4,0.98) | 0.40 |  | 0.19 (-0.28,0.67) | 0.42 |  | -0.00 (-0.01,0.01) | 0.99 |
| Years Since HIV Diagnosis |  | 0.13 (-0.47,0.72) | 0.67 |  | -0.39 (-0.86,0.08) | 0.10 |  | -0.29 (-0.98,0.4) | 0.40 |  | -0.19 (-0.67,0.28) | 0.42 |  | 0.00 (-0.01,0.01) | 0.99 |
| Age at ART Initiation |  | -0.42 (-1.06,0.22) | 0.20 |  | 0.34 (-0.20,0.87) | 0.21 |  | 0.04 (-0.74,0.81) | 0.92 |  | 0.02 (-0.52,0.56) | 0.94 |  | -0.001 (-0.02,0.01) | 0.94 |
| Years on ART |  | 0.42 (-0.22,1.06) | 0.20 |  | -0.34 (-0.87,0.20) | 0.21 |  | -0.04 (-0.81,0.74) | 0.92 |  | -0.02 (-0.56,0.52) | 0.94 |  | 0.001 (-0.01,0.02) | 0.94 |
| CD4+ Count at Enrollment |  | -0.005 (-0.01,0.004) | 0.29 |  | 0.003 (-0.004,0.01) | 0.42 |  | 0.00 (-0.01,0.01) | 0.99 |  | -0.001 (-0.01,0.01) | 0.69 |  | 0.00 (-0.00,0.00) | 0.92 |
| CD4+ Count after ART |  | -0.002 (-0.02,0.01) | 0.82 |  | 0.01 (0,0.02) | 0.14 |  | -0.005 (-0.02,0.02) | 0.64 |  | 0.01 (-0.01,0.02) | 0.41 |  | 0.00 (-0.00,0.00) | 0.46 |
| Age at HIV Diagnosis: when participants tested positive for HIV; CD4+ count at enrollment and after ART represents CD4+ levels (cells/uL) upon entering the study and beginning ART treatment, respectively | | | | | | | | | | | | | | | |
| Primary models are adjusted for: | | | | | | | | | | | | | | | |
| Model 1: age (years) + sex + past smoking status (never or ever) + education level + cell-type composition (NK cell, neutrophil, B cell, monocyte, CD8+ and CD4+ T cell) | | | | | | | | | | | | | | | |
| Model 2: Model 1 + Daily dietary intake average (servings) | | | | | | | | | | | | | | | |
| Model 3: Model 1 + SF-36; SF-36 uses a 36-item questionnaire to assess perceived quality of life | | | | | | | | | | | | | | | |
| Model 4: Model 1 + total physical activity (minutes of moderate/vigorous work, commute, and recreational/leisure activity) | | | | | | | | | | | | | | | |
| Secondary models are performed using the same covariates with no adjustment for cell-type composition | | | | | | | | | | | | | | | |
| Significance taken at *p <*0.05 | | | | | | | | | | | | | | | |

| **Supplementary Table 2. Association between epigenetic aging and quality of life/lifestyle behaviors** | | | | | | | | | | | | | | | | |  |
| --- | --- | --- | --- | --- | --- | --- | --- | --- | --- | --- | --- | --- | --- | --- | --- | --- | --- |
|  |  |  |  |  |  |  |  |  |  |  |  |  |  |  |  | | |
|  |  | Horvath Clock | |  | Hannum Clock | |  | PhenoAge Clock | |  | GrimAge Clock |  |  | DunedinPACE | |  |  |
| Variable |  | β-Estimate (95% CI) | *p-*value |  | β-Estimate (95% CI) | *p-*value |  | β-Estimate (95% CI) | *p-*value |  | β-Estimate (95% CI) | *p-*value |  | β-Estimate (95% CI) | *p-*value | | |
| *Primary Model* |  |  |  |  |  |  |  |  |  |  |  |  |  |  |  | | |
| Daily Dietary Intake Average |  | 0.71 (-4.71,6.12) | 0.79 |  | -1.44 (-5.75,2.86) | 0.49 |  | 1.05 (-5.27,7.37) | 0.73 |  | -0.88 (-5.59,3.83) | 0.70 |  | **0.12 (0.03,0.22)** | **0.01** | | |
| Weekly Dietary Intake |  | -0.00 (-0.51,0.51) | 1.00 |  | -0.07 (-0.47,0.34) | 0.74 |  | 0.05 (-0.54,0.65) | 0.85 |  | 0.14 (-0.30,0.58) | 0.50 |  | -0.01 (-0.02,0.00) | 0.09 | | |
| SF-36 |  | -0.03 (-0.15,0.10) | 0.69 |  | 0.07 (-0.06,0.19) | 0.28 |  | -0.06 (-0.22,0.10) | 0.45 |  | 0.03 (-0.07,0.13) | 0.58 |  | -0.00 (-0.00,0.00) | 0.50 | | |
| Total Physical Activity |  | 0.00 (-0.00,0.00) | 0.16 |  | 0.00 (-0.00,0.00) | 0.13 |  | 0.00 (-0.00,0.00) | 0.59 |  | -0.00 (-0.00,0.00) | 0.07 |  | -0.00 (0.00,0.00) | 0.64 | | |
| *Secondary Model* |  |  |  |  |  |  |  |  |  |  |  |  |  |  |  | | |
| Daily Dietary Intake Average |  | 0.65 (-4.35,5.65) | 0.79 |  | -1.7 (-5.55,2.16) | 0.37 |  | 1.91 (-3.44,7.27) | 0.47 |  | 0.79 (-3.56,5.15) | 0.71 |  | **0.15 (0.05,0.24)** | **0.01** | | |
| Weekly Dietary Intake |  | -0.01 (-0.46,0.44) | 0.96 |  | -0.05 (-0.4,0.3) | 0.78 |  | 0.04 (-0.45,0.53) | 0.87 |  | -0.02 (-0.41,0.37) | 0.93 |  | **-0.01 (-0.02,0.00)** | **0.03** | | |
| SF-36 |  | 0.01 (-0.12,0.13) | 0.92 |  | 0.04 (-0.06,0.14) | 0.41 |  | -0.10 (-0.23,0.04) | 0.16 |  | -0.04 (-0.14,0.07) | 0.49 |  | **-0.003 (-0.01,0.00)** | **0.05** | | |
| Total Physical Activity |  | 0.001 (0.00,0.00) | 0.07 |  | 0.001 (0.00,0.00) | 0.11 |  | -0.00 (0.00,0.00) | 0.77 |  | -0.001 (0.00,0.00) | 0.01 |  | 0.00 (0.00,0.00) | 0.06 | | |
| Primary models are adjusted for: age + sex + past smoking status + education level + estimated cell type composition | | | | | | | | | | | | | | | | |  |
| Secondary models are adjusted for: age + sex + past smoking status + education level | | | | | |  |  |  |  |  |  |  |  |  |  | | |
| Daily Dietary Intake Average: average number of servings of fruits/vegetables consumed per day | | | | | | | | | | | | | | | | |  |
| SF-36: self-administered 36-item questionnaire to assess perceived overall quality of life | | | | | | | | | | | | | | | | |  |
| Total Physical Activity: the number of minutes of work-related (moderate, vigorous), commuting-related (bike, walk), and recreational/leisure time (moderate, vigorous) physical activity | | | | | | | | | | | | | | | | |  |
